# Genetic Differentiation and Delimitation between Ecologically Diverged *Populus euphratica* and *P. pruinosa*

**DOI:** 10.1371/journal.pone.0026530

**Published:** 2011-10-19

**Authors:** Juan Wang, Yuxia Wu, Guangpeng Ren, Qiuhong Guo, Jianquan Liu, Martin Lascoux

**Affiliations:** 1 Molecular Ecology Group, State Key Laboratory of Grassland Farming System, Lanzhou University, Lanzhou, Gansu, China; 2 Laboratory of Evolutionary Genomics, CAS-MPG Partner Institute for Computational Biology, Chinese Academy of Sciences, Shanghai, China; 3 Program in Evolutionary Functional Genomics, Evolutionary Biology Centre, Uppsala University, Norbyvägen, Uppsala, Sweden; American Museum of Natural History, United States of America

## Abstract

**Background:**

The fixed genetic differences between ecologically divergent species were found to change greatly depending on the markers examined. With such species it is difficult to differentiate between shared ancestral polymorphisms and past introgressions between the diverging species. In order to disentangle these possibilities and provide a further case for DNA barcoding of plants, we examine genetic differentiation between two ecologically divergent poplar species, *Populus euphratica* Oliver and *P. pruinosa* Schrenk using three different types of genetic marker.

**Methodology/Principal Findings:**

We genotyped 290 individuals from 29 allopatric and sympatric populations, using chloroplast (cp) DNA, nuclear (nr) ITS sequences and eight simple sequence repeat (SSR) loci. Three major cpDNA haplotypes were widely shared between the two species and between-species cpDNA differentiation (*F_CT_*) was very low, even lower than among single species populations. The average SSR *F_CT_* values were higher. Bayesian clustering analysis of all loci allowed a clear delineation of the two species. Gene flow, determined by examining all SSR loci, was obvious but only slightly asymmetrical. However, the two species were almost fixed for two different nrITS genotypes that had the highest *F_CT_*, although a few introgressed individuals were detected both in allopatric and sympatric populations.

**Conclusions:**

The two species shared numerous ancestral polymorphisms at cpDNA and a few SSR loci. Both ITS and a combination of nuclear SSR data could be used to differentiate between the two species. Introgressions and gene flow were obvious between the two species either during or after their divergence. Our findings underscore the complex genetic differentiations between ecologically diverged species and highlight the importance of nuclear DNA (especially ITS) differentiation for delimiting closely related plant species.

## Introduction

Genetic variants are frequently shared between closely related tree species of diverse genera, including *Picea* [1], *Pinus* [2], *Abies* [3], *Quercus* [4], *Betula* [5], *Eucalyptus* [6]–[8] and *Populus* [9],[10]. Allele sharing and its variation among loci can result from three factors that are not exclusive but are difficult to distinguish: incomplete lineage sorting of shared ancestral polymorphisms, introgressive hybridization after the initial interspecific divergence, and homoplasy [7], [11]–[13]. Furthermore, the shared variation between species differs greatly according to the genetic markers examined. For example, in conifers, chloroplast DNA variants tend to be shared less frequently than mitochondrial DNA ones [14]–[15], and in many angiosperm genera, chloroplast DNA is less effective in delimiting species boundaries or intraspecific units than nuclear DNA [16]–[17]. Three main hypotheses have been put forward to explain the variation observed in allele sharing among the different types of markers. First, different markers have different ploidy levels and inheritance modes and thus different effective population sizes. For example, in angiosperms, nuclear DNA is diploid and bi-parentally inherited, while chloroplast (cp) and mitochondrial (mt) DNA are haploid and maternally inherited. Thus, in the case of poplars, which are dioecious, the effective population sizes of cytoplasmic markers will be one fourth of the effective population size of nuclear markers. When the sex-ratio is even, or in monoecious species that exhibit random mating, lineage sorting will take longer for nuclear than for cytoplasmic genes because of their larger effective population size [18]–[19]. However, empirical observations in numerous angiosperm species do not support these expectations [8], [11], [16]–[17]. Interestingly, the different types of markers are not affected in the same way by a biased sex-ratio, a common feature of Salicaceae [20]. A female biased sex-ratio will decrease the effective population size for nuclear markers but will, proportionally, increase the effective population size of cytoplasmic markers that are maternally inherited. Therefore, the difference between the nuclear lineage sorting time and that for the cytoplasmic genes in Salicaceae is not clear. Second, the number of fixed sites between species is positively related to the rate of gene flow of the markers within the species. If a species is strongly structured at some genetic markers but less so at others, then the coalescence time will differ accordingly, resulting in more time to reach reciprocal monophyly at markers experiencing low rates of gene flow within species than at markers experiencing high rates of gene flow [21]. Recent modeling and empirical studies consistently suggest that the introgressed alleles will be rapidly diluted by high gene flow among populations of the hybridizing species [22]. Finally, it is possible that most ecologically diverged species differentiate only with respect to speciation genes that are associated with species-specific adaptations or linked loci [23]–[24] while at the others, the interspecific differentiation is extremely low. However, in combination, the minimal differentiation of these multiple loci can delimit two species well, even when cytoplasmic markers with their small effective population sizes remain un-differentiated [25]. This approach may be useful for disentangling introgressions from ancestral polymorphisms at certain loci. In addition, in plants, nuclear ribosomal internal transcribed spacer (nrITS) sequences, which comprise multiple loci but with concerted evolution, may have undergone faster inter-specific differentiation than other nuclear loci, with the exception of speciation genes and linked fragments [26]. Thus, such fragments are likely to be well suited to delimiting and barcoding closely related species resulting from ecological divergences. However, all these hypotheses need further testing, especially in plants, based on genetic comparisons of closely related species using different markers.

Here, we use three different markers to examine genetic differentiation and introgression between two closely related poplar species that occur in riparian deserts, namely the Euphrates poplar (*Populus euphratica* Oliver) and the desert poplar (*P. pruinosa* Schrenk). *P. euphratica* and *P. pruinosa* are the two most economically important species of the section *Turanga* (Afro-Asian poplars), which also includes the Kenyan poplar (*P. ilicifolia*) [27]. Although the exact phylogenetic position of this section remains unclear, these two species are always phylogenetically closely related to each other (Figure S1 and S2). *P. euphratica* has a large discontinuous distribution, extending from Morocco to China, spanning 115° of longitude and 34° of latitude. *P. pruinosa* has a more restricted distribution and grows in alluvial oasis communities in northwest China, Kazakstan, Tajikistan, Turkmenistan, and Uzbekistan. Both play an important role in maintaining local arid ecosystems [28]. They can grow in deserts with extremely hot and dry summers, while other congeners are known to be drought-sensitive [29]–[31]. In fact, *P. euphratica* is an important model species for examining abiotic resistances in trees, e.g. responses to salinity or drought stress [32]–[35]. The two species can easily be distinguished from each other based on leaf morphology. *P. euphratica* has two types of glabrous or almost glabrous leaves [27]: young individuals and twigs from the basal part of old trees bear lanceolate leaves while leaves from old twigs of mature trees are oval, extravagant oval, broad-triangular or dentate broad-ovate. In contrast, all leaves of *P. pruinosa* are ovate or kidney-shaped and bear thick hairs (Figure S3). Although both species occur in deserts, *P. euphratica* prefers dry and salty deserts with a low underground water level, whereas *P. pruinosa* grows in environments with a high underground water level near ancient or extant rivers. These two species, therefore, provide a good model to study genetic differentiation and cryptic introgression during ecological speciation.

We genotyped 290 individuals from 29 allopatric and sympatric populations of the two species, using cpDNA, nrITS sequence data and polymorphisms from eight simple sequence repeat (SSR) loci. Together these three markers include different ploidy levels, inheritance modes, and mutation models and rates. In poplars, chloroplast DNA is mainly maternally inherited and dispersed via seeds, while nuclear DNA is bi-parentally inherited and dispersed via both pollen and seeds. In addition, pollen is wind dispersed while seeds are dispersed by both wind and water [27]. Our study was designed to combine information from these different markers to address the following questions: (i) Is allele sharing more important at chloroplast DNA markers than nuclear DNA markers? If so, which factors contribute to this difference? (ii) How do these markers delimit the two species? Does a combination of multiple nuclear SSR loci define the two species better than nrITS or cpDNA? (iii) Did nuclear introgression occur? Where did hybridization occur, in sympatric populations, allopatric populations or both?

## Results

### cpDNA Sequence Variation

**Figure 1:**
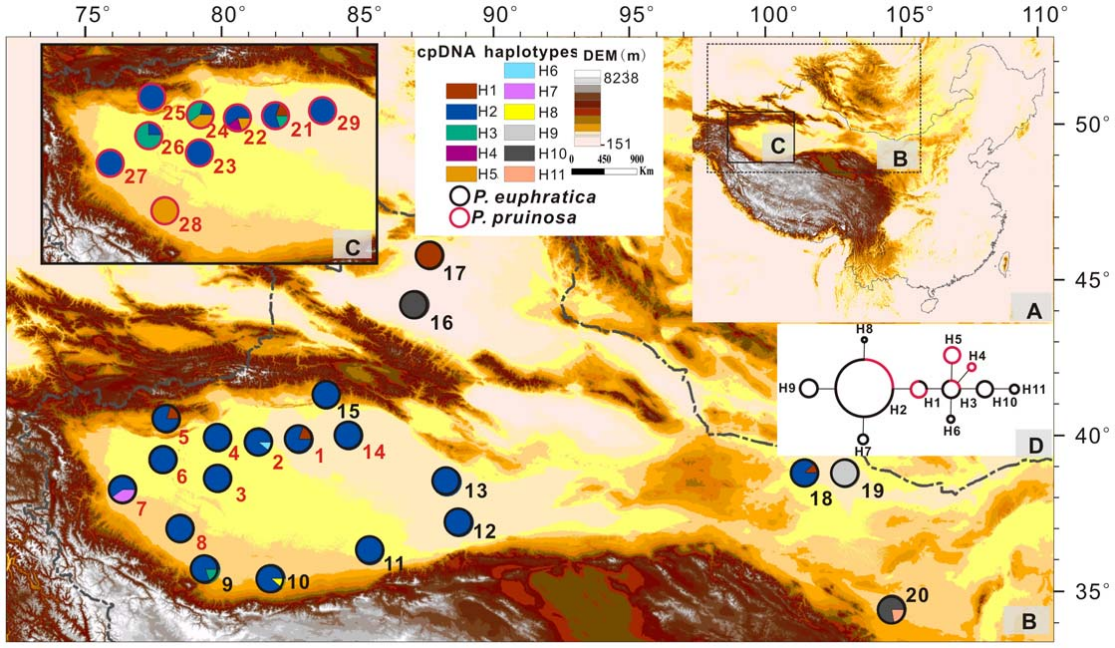
Sampling sites and chlorotype frequencies in the examined populations of the two poplar species. (A) Map of China. (B) Chlorotype frequencies of *P. euphratica*. (C) Chlorotype frequencies of *P. pruinosa*. (D) Network of the chlorotypes. Populations marked in red indicate the sites where the two species grow sympatrically. Circle size is proportional to chlorotype frequency, with the largest circle representing the most common haplotype.

Four indels and five substitutions were detected in the *trn*V and *trn*L fragments, and these resolved into 11 haplotypes (H1–H11, Table S2) among the 290 trees surveyed. The three most frequent haplotypes (H1, H2 and H3) were shared between the two species and were located near the center of the chlorotype network; haplotypes derived from these three core haplotypes were species-specific (Figure 1). Phylogenetic analysis of all chlorotypes, with *P. trichocarpa* used as an outgroup, showed that phylogenetic relationships among chlorotypes were poorly resolved (Figure S4). The total genetic diversity (*H*_T_) was clearly higher than the diversity within populations (*H*_S_) for both species. In addition, *H*_T_ and *H*_S_ were higher in *P. pruinosa* than in *P. euphratica*. However, Nei's fixation index, *G*_ST_ was higher in *P. euphratica* than in *P. pruinosa*. Phylogeographic structure was only obvious in *P. pruinosa* (*N*_ST_>*G*_ST_, *P*<0.05, Table 1). AMOVA analyses suggested that 14.86% of the total cpDNA variation could be attributed to variation between species (*F_CT_* = 0.1486, Table 2), whereas 53.74% could be attributed to variation among populations within species (Table 2). The intraspecific differentiation among different localities (populations) was obviously larger than that between species. Only 20.0% or 22.0% of the sampled individuals in *P. euphratica* or *P. pruinosa*, respectively, carried species-specific chlorotypes while the rest shared common ones (Figure S6).

**Table 1:** Estimates of average gene diversity within populations (*H_S_*), total gene diversity (*H_T_*), inter-population differentiation (*G_ST_*), and the number of substitution types (*N_ST_*) (mean±SE in parentheses) within the total distributional range (based on cpDNA haplotypes) calculated with *PERMUT*, using a permutation test with 1,000 permutations.

Species	*H*_S_	*H*_T_	*G*_ST_	*N*_ST_
*P. euphratica*	0.168 (0.0556)	0.447 (0.1134)	0.625 (0.1406)	0.556 (0.1578)
*P. pruinosa*	0.300 (0.1213)	0.590 (0.1232)	0.492(0.1809)	0.590 (0.1777)**
**Pooled**	0.226 (0.0522)	0.429 (0.0879)	0.473 (0.1131)	0.664 (0.2289)

**Indicates that *N*_ST_ is significantly different from *G*_ST_ (*P<*0.001).

**Table 2:** Analyses of molecular variance (AMOVA) in the two poplar species based on cpDNA haplotypes, ITS genotypes and SSR markers.

Grouping of regions	Source of variation	d.f.	SS	VC	Percentvariation	Fixation index
**cpDNA haplotypes**						
All samples	Among species	1	52.972	0.3262	14.86	*F_CT_* = 0.1486
	Among populations within species	27	337.028	1.1794	53.74	*F_ST_* = 0.6861
	Within populations	261	179.800	0.6889	31.39	*F_SC_* = 0.6313
*P .euphratica*	Among populations	19	92.050	0.4465	54.02	*F_ST_* = 0.5402
	Within populations	180	68.400	0.3800	45.98	
*P. pruinosa*	Among populations	8	244.978	2.9247	68.02	F_ST_ = 0.6802
	Within populations	89	111.400	1.3753	31.98	
**ITS genotypes**						
All samples	Among species	1	289.944	1.157	84.34	*F_CT_* = 0.8434
	Among populations within species	27	72.221	0.129	9.44	*F_ST_* = 0.9378
	Within populations	551	47.000	0.085	6.22	*F_SC_* = 0.6029
**SSR markers**						
All samples	Among species (average)	1	186842.932	725.670	30.20	*F_CT_* = 0.3020
	Among populations within species	27	180266.686	263.124	10.95	*F_ST_* = 0.4115
	Within populations	551	779147.550	1414.061	58.85	*F_SC_* = 0.1569
*P .euphratica*	Among populations	19	125619.853	255.271	14.49	*F_ST_* = 0.1450
	Within populations	380	572340.350	1506.159	85.51	
*P. pruinosa*	Among populations	8	51896.500	273.412	21.16	*F_ST_* = 0.2116
	Within populations	171	174217.800	1018.818	78.84	

d.f., degrees of freedom; SS, sum of squares; VC, variance components; *F_ST_*, variance among populations; *F_SC_,* variance among populations within groups; *F_CT_,* variance among groups relative to total variance.

### ITS Sequence Variation

**Figure 2:**
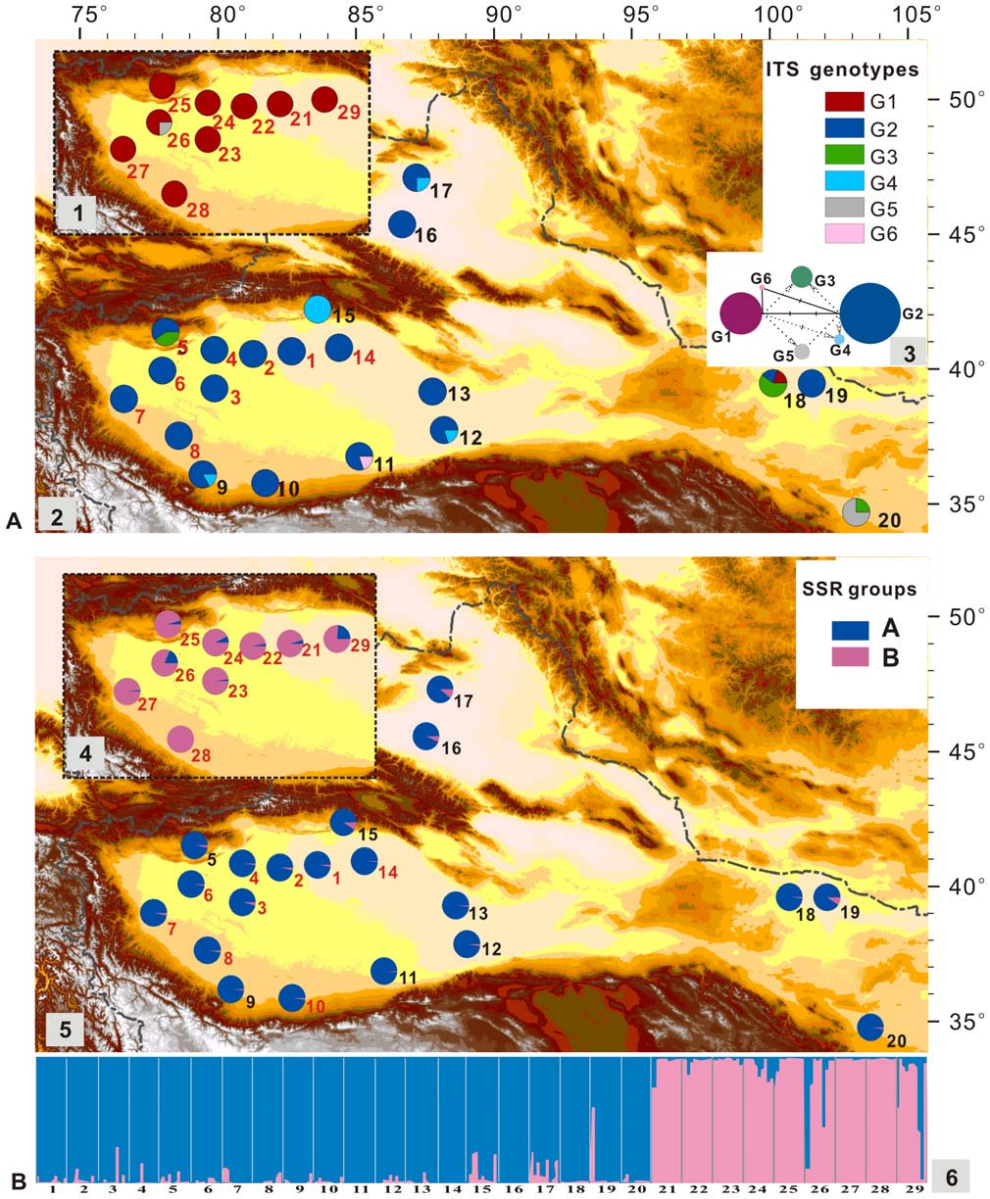
The distributions of ITS genotype and SSR genetic groups. A: 1. ITS genotype frequencies in *P. pruinosa*; 2. ITS genotype frequencies in *P. euphratica*; 3. Network relationships of ITS genotypes. B: 4. One SSR genetic group in *P. pruinosa*; 5. The other SSR group in *P. euphratica*; 6. Proportion of inferred coancestry, determined by Bayesian clustering analysis, for the two species based on all SSR loci (*K* = 2) (equating to the geographical distributions shown in 4 and 5).

Two common ITS genotypes (G1 and G2), which differed by three nucleotide substitutions, were identified (Table S3). G1 was restricted to *P. pruinosa*, while G2 occurred exclusively in *P. euphratica* (Figure 2A). Three other genotypes (G3, G5 and G6) were obvious heterozygotes derived from hybridizations between genotypes G1 and G2 (Table S3). Two different copies were recovered when the positive clones of the PCR products of G3, G5 and G6 were cloned and sequenced (Table S3). One copy of these three genotypes was identical to G1 (or G2) and the other was similar to G2 (or G1), but had fixed species-specific mutations (Table S4). Although G4 was pure when all corresponding PCR products were directly sequenced, it was found to be similar to G2 with one G1-specific mutation fixed (Figure 2A). This genotype may have also derived from hybridization between G1 and G2 and past concerted evolution in multiple ITS loci may have created this “homozygote”.

If we excluded the four putative hybrid genotypes (G3, G4, G5 and G6), 93.4% and 98.0% of the individuals had the species-specific genotypes (G1 or G2) in sampled populations of *P. euphratica* and *P. pruinosa*, respectively (Figure S6). The four putative hybrid genotypes were widely distributed in both allopatric and parapatric populations of *P. euphratica* but were found in only one parapatric population of *P. pruinosa*. According to the AMOVA analyses, 84.34% of the total ITS variation could be attributed to variation between species (Table 2, *F_CT_* = 0.8434).

### Genetic Differentiation at the Microsatellite Loci

We used polyacrylamide denaturing gel and silver staining to identify genetic polymorphisms at each locus. SSR polymorphisms are probably underestimated by this technique, since allelic differences caused by a small number of repeats may go undetected. If these missed mutations are recent and tend to be species-specific it is possible to overestimate the similarity between the two species. In total, 97 alleles were identified across the two species. In *P. euphratica*, the number of alleles ranged from 8 to 13 with an average of 11.13 alleles per locus while in *P. pruinosa* the number of alleles ranged from 6 to 13 with an average of 8.88 alleles per locus. *H_o_* ranged from 0.83 to 1.00 with an average of 0.95 in *P. euphratica* and from 0.16 to 1.00, with an average of 0.65 in *P. pruinosa* (Table S5). The average expected heterozygosity (*H*_e_) was lower in *P. pruinosa* than *P. euphratica* (0.75vs0.84). The same trend was shown in Table S6. Across the two species, *F*_is_ values ranged from −0.3435 to 0.0063, with an average value of −0.1946 while *F*_it_ values varied from −0.3845 to 0.1752 with a mean value of −0.0169, suggesting an overall excess of heterozygotes.

**Figure 3:**
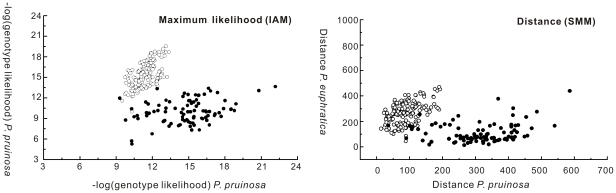
Individual assignment based on maximum likelihood analyses of nuclear SSR alleles. The methods were based on allele frequencies under an infinite alleles model (IAM) (left) and a distance method (right) using differences in microsatellite allele lengths under a stepwise mutation model (SMM). The filled dots represent *P. pruinosa*, and the open circles *P. euphratica*.

AMOVA analyses indicated that 30.20% of the genetic variation occurred between species (average *F_CT_* = 0.3020), whereas only 10.95% of the variation was partitioned among populations within species. However, at each locus, inter-specific differentiation varied greatly (from *F_CT_* = −0.0167 to *F_CT_* = 0.6035, Table S4). Stepwise mutation model (SMM) and infinite allele model (IAM) analyses yielded similar results, with two largely non-overlapping groups corresponding to the morphological clustering of the two species (Figure 3). Because *L*(*K*) continued to increase after *K* = 2 clusters (Figure S5A), the most suitable *K* value for the STRUCTURE analyses was not unexpected given the presence of additional substructure at lower hierarchical levels. However, the genetic structure based on arbitrary assignments of all sampled individuals into two groups was largely consistent with morphological delimitations and the best Δ*K* value was found to be 2 (Figure S5B). Under this two-group assignment, assuming we interpreted admixed individuals as reflecting introgression, introgression occurred in both directions but was slightly more common from *P. euphratica* to *P. pruinosa* (Figure 2B). The introgression in the opposite direction, from *P. pruinosa* to *P. euphratica*, occurred in both allopatric and parapatric populations. If the individuals with more than 5% microsatellite polymorphisms of the other species were treated as having introgressed, 96.2% or 92.8% of the sampled individuals in *P. euphratica* or *P. pruinosa* were pure, based on their species-specific microsatellite compositions (Figure S6).

The directionality of gene flow between the two species was examined further and an estimation made with MIGRATE using the pooled data for each species (Table 3). The estimated effective population size of *P. euphratica* (1475) was larger than that of *P. pruinosa* (982). If all shared polymorphisms were assumed not to be the result of incomplete lineage sorting of ancestral polymorphisms, the gene flow was estimated to be slightly asymmetrical with higher gene flow from *P. euphratica* to *P. pruinosa* than in the opposite direction.

**Table 3:** Effective population sizes (*N_e_*) in *P. euphratica* and *P. pruinosa*, and effective migration rates (*N_e_*m) between species, estimated by coalescent theory and a maximum-likelihood-based approach.

Species	θ	M_12_	M_21_	*N*_e_	2*N*_e_m_12_	2*N*_e_m_21_
*P. euphratica*	5.8998	-	2.2203	1475	-	6.55
	5.6816–6.1276	-	2.0636–2.3843	1420–1532	-	5.86–7.31
*P. pruinosa*	3.9280	3.8661	-	982	7.59	-
	3.7461–4.1290	3.5566–4.1685	-	937–1032	6.66–8.61	-

*N*_e_, effective population size.

μ, mutation rate (μ = 10^−3^per gamete per year).

2*N*_e_m_12_, the effective number of migrants from *P. euphratica* to *P. pruinosa* per generation (10 years).

2*N*_e_m_21_, the effective number of migrants from *P. pruinosa* to *P. euphratica* per generation (10 years).

The range estimates given below each value are the 95% confidence limits.

## Discussion

The genetic analysis of species boundaries between the two sister species *P. euphratica* and *P. pruinosa* led to very different conclusions depending on the type of markers considered. Although some of the rare cpDNA chlorotypes were species-specific, the three most common haplotypes (H1, H2 and H3) were widely shared across the species (Figure 1). We found that intraspecific differentiation based on different localities was higher than that between species. In contrast, the two most common genotypes at the nrITS were species-specific and limits between the two clusters identified by STRUCTURE analysis, based on a combination of SSR loci, corresponded neatly to the species limits. Interspecific differentiation (*F*_CT_) varied greatly between the three types of genetic marker (Table 2) and between different SSR loci (Table S4). In addition, this differentiation also shifted between SSR loci, and the average differentiation was lower than that according to ITS marking. All these findings support recent predictions [21]–[26] and are, in part, consistent with the limited number of studies involving one or two similar genetic markers in plants [16], [17]. In addition, our assessment of nuclear markers of the two species at the population level also revealed cryptic introgressions during or following the divergence of the two species.

### Ancestral polymorphisms

Alleles shared between species may originate from: (i) the ancestral population of the two species (incomplete lineage sorting); (ii) gene flow between the two species after their separation; or (iii) homoplastic mutation at the same sites in two species with different ancestors. Important factors, therefore, are the split time, the effective population sizes, the rate of gene flow and the mutation rate and process. In addition, natural selection may play a role in the establishment of barriers to gene flow. In angiosperms, substitution rate tends to be lower for chloroplast than nuclear DNA (with a ratio of 1∶5) [36]. However, the difference seems to be smaller between cpDNA and nrITS or nuclear SSR data, although it should be acknowledged that the available data do not allow a straightforward comparison [37]. Thus, the poor discriminatory power of cpDNA could simply be due to a lack of polymorphism. In the present case this seems, however, unlikely to be the main cause since the number of segregating sites at ITS between the two species was even lower than for cpDNA. Similarly, homoplasy does not appear to be a plausible explanation since it is most pervasive at SSR loci, which, even so, provide good differentiation of the two species at some loci (for example, *F_CT_* = 0.6035; Table S4) or a combination of all loci (Table 2).

In fact, the central position of the shared chlorotypes in the species network obviously suggests the widespread occurrence of incomplete sorting of ancestral polymorphisms between the two species. Although we cannot rule out the possibility that gene flow may have partly caused the observed shared chlorotypes, ancestral polymorphisms appear to have contributed greatly to the low cpDNA differentiation between the two poplar species. This is further supported by the fact that intraspecific cpDNA differentiation among localities was larger than that between species (Table 2). In plants, cpDNA is maternally inherited and all DNA fragments are linked as a single unit, it is likely that the interspecific differentiation in such a single cytoplasmic DNA is comparable to that of a single nuclear locus [25]. In fact, we found that the interspecific differentiation at some SSR loci was lower than that of the cpDNA (Table S4). At these loci, it is probable that these two species also shared numerous ancestral polymorphisms; however, a combination of all SSR loci produced better differentiation (Table 2) as suggested previously [25]. However, at certain loci, the interspecific differentiation increased greatly (Table S4). These SSR loci may have been linked to speciation genes or those related to the local adaption of the two species and, therefore, may have experienced accelerated interspecific differentiation. It is interesting that we detected the highest interspecific differentiation on the basis of ITS marking (Table 2). The detailed mechanism of the rapid lineage sorting between recently diverged species associated with this nuclear DNA fragment (comprising multiple loci with concerted evolution) remains unknown. However, a combination of at least two different factors may have led to a more restricted gene exchange between the species and a higher rate of lineage sorting associated with this ITS fragment and other nuclear loci (for example, some SSR loci observed here).

First, a high rate of gene flow within individual species will increase the rate of lineage sorting between different species [2] and thereby reduce the level of introgression [22], [38]. In poplars, cpDNA is maternally inherited and dispersed by seeds while nDNA is biparentally inherited and dispersed by both seeds and pollen [27]. Although both the seed and the pollen of poplars are wind dispersed, the dispersal distance of seeds is likely to be shorter than that of pollen [9]. Therefore, it is highly likely that the relatively low rate of intraspecific gene flow could have reduced the sorting of cpDNA ancestral polymorphisms [2], [19], [21], [22], [38]–[40]. Despite this, we found that cpDNA had several species-specific variants for both *P. euphratica* and *P. pruinosa* (Figure 1), suggesting that these cpDNA variants may progressively replace the ancestral polymorphisms and become monophyletic after a longer period of divergence. Second, it is possible that the difference in effective population sizes between cpDNA and nuclear DNA is not as large as the inheritance of the markers would suggest once all demographic factors have been taken into account. In particular, Salicaceae often exhibit biased sex ratios. For example, Rottenberg et al. [20] sexed individuals in three populations of *P. euphratica:* females accounted for 57.1, 100 and 0% of the populations, respectively. A female-biased sex ratio would simultaneously increase the effective population size for cpDNA (cp*N*_e_ will tend towards the number of females, *N_f_*) and reduce *N*_e_ at nuclear markers (the nuclear *N*_e_ will tends towards 4 since *N*_e_ = 4*N*_m_ *N*_f_/(*N*_m_+*N*_f_) where *N_m_* and *N_f_* are the number of males and females, respectively). In the case of a very strongly biased female sex-ratio, it is even possible to have a larger cpDNA *N*_e_ than nuclear *N*_e_. While such an extreme case is unlikely, a female biased-sex ratio would nonetheless tend to diminish the difference in effective population size between the two types of marker.

### Introgressions between two species

Despite the greater differentiation at nDNA than cpDNA between the two species, the data suggest the occurrence of ancient gene flow between them. Our survey of nrITS variation revealed two major genotypes, G1 and G2, which separately characterize the two species. The other four genotypes are recombinants between these two genotypes and indicate ancient introgression; this is because individuals carrying those genotypes have species-specific taxonomic traits and show no signs of intermediate morphology. One of these recombinant genotypes is found in a few individuals of population 26 of *P. pruinosa*, which grows close to *P. euphratica* but the others (G4, G5 and G6) are distributed in both allopatric and parapatric *P. euphratica* populations (Figure 2A). The presence of these genotypes in allopatric populations located far away from the current distribution of *P. pruinosa* could reflect long distance dispersal of *P. pruinosa* or, perhaps more likely, the changing distribution of the two species over time. These regions have become drier over recent centuries, rivers have disappeared, underground waters have become deeper [41] and the environment is now less suitable for *P. pruinosa* [42]. Since individuals of *P. euphratica* can live more than 200 years [43], [44], some of the recombinants observed today may originate from the original admixed population. In addition, there are more hybrid individuals of *P. euphratica* than of *P. pruinosa* (Figure 2A, 5). This suggests asymmetrical gene flow, which is more consistent with the flowering stages of both species. These two species are dioecious with the males flowering earlier than the females; in addition *P. euphratica* flowers earlier than *P. pruinosa*, although their flowering periods largely overlap [45]. The later-developing ovules of *P. euphratica* are thus more likely to be pollinated by pollen from *P. pruinosa* which will still be in its early male flowering stage.

However, based on the SSR loci, we found that gene flow seems to be slightly asymmetric in the opposite direction, with higher gene flow from *P. euphratica* to *P. pruinosa*. This inconsistency probably results from three factors. First, gene flow calculated on the basis of SSR loci includes all polymorphisms shared between the two species. Some of these polymorphisms may have originated from incomplete lineage sorting of ancestral lines. Therefore, gene flow determined in this way may be biased upward. Second, the gene flow detected by SSR loci may have included all interspecific genetic exchanges since the species diverged, comprising both during-speciation and post-speciation gene flow. However, the hybridization signals detected by ITS marking may, in fact, indicate the second stage of introgressions after initial divergence of the species [46]. Third, the opposite asymmetric gene flow detected by ITS may further result from the relative abundance of two species during the second introgressions. The direction of the backcrosses was found to occur predominantly towards the more numerous species [47]. According to our field exploration and the previous surveys, *P. euphratica* has a wider distribution and larger population size than *P. pruinosa* [28]. Finally, it should be noted that such introgressions must have been ancient, based on the current geographical distributions of the two species.

Two main conclusions emerge in this study. First, the present and previous studies [9] confirm the labile nature of species barriers in Salicaceae, although most species are morphologically distinct. Our study, however, also indicates that a large part of the allele sharing that we see today reflects incomplete lineage sorting and ancient introgression rather than ongoing gene flow. Incidentally, it also illustrates how examining markers with different inheritance and mutation processes can provide new insights. Second, our results and those of previous studies on closely related angiosperm species suggest that cpDNA is less effective in delineating species boundaries than nuclear DNA markers [8], [11]. Nuclear DNA, especially if many markers are used or if those used are related to species-adaptations will be better than cytoplasmic DNA for barcoding closely related species that have undergone ecological divergences [25], [48]. This is extremely relevant to the widespread use in plant taxonomy of DNA barcodes [49]–[51]. If barcoding is to be based on a single marker, nrITS appears to be a good candidate DNA for barcoding closely related species [26], [52].

## Materials and Methods

### Sampled Populations

*P. pruinosa* always grows sympatrically with *P. euphratica*, but the latter species has a much wider distribution. We sampled 29 populations of the two species. Both species were sampled at nine sympatric sites (a total of 18 populations) and 11 populations of *P. euphratica* were also collected from allopatric regions (Figure 1, Table S1). Because our main aim was to examine genetic differentiation and introgression (as opposed to hybridization), we sampled only typical individuals of each species. At the sympatric sites, we avoided collecting material from the contact zone between the two species, although we did not find distinct hybrids with intermediate morphology. For each population, we randomly sampled 10 individuals. Those individuals were at least 100 m apart, in order to minimize the possibility of collecting identical clones, clonal reproduction being common in *P. euphratica* and *P. pruinosa* [53]. Because natural populations of both species shrank in the past years due to the anthropic activities [28], such a sampling strategy may have underestimated the actual genetic diversity of each species. However, this will not affect our main aims to examine the genetic differentiation and delimitations of two species. Fresh leaves were dried and stored in silica gel. The latitude, longitude and altitude of each collection site were recorded using an Extrex GIS unit (Garmin, Taiwan).

### Molecular Markers

Genomic DNA was isolated from each tree using the modified hexadecetyltrimethyl ammonium bromide (CTAB) procedure [54]. Fourteen pairs of primers for cpDNA regions (*rp*l16f-r, *mat*K, *rbc*L, *trn*L-F, *trn*H-*psb*A, *rps*4, *trn*G-*trn*M, *psb*A, *trn*A, *rpo*B-*trn*C, *rpo*B *trn*L, *trn*V and *psa*A) [55]–[57] were used to detect possible inter-specific variation across eight individuals of the two species sampled far apart. Only two pairs of primers (*trn*L and *trn*V) revealed inter-individual variation. These two cpDNA fragments and the nrITS (ITS1-5.8S-ITS2) region [58] were then amplified and sequenced in all individuals. Polymerase chain reaction (PCR) was performed in a 25 µL volume containing: 10–40 ng plant DNA, 50 mM Tris-HCI, 1.5 mM MgCl_2_, 250 µg/mL BSA, 0.5 mM dNTPs, 2 µM of each primer and 0.75 unit of Taq polymerase. All reactions were performed using the following thermal profile: 7 min at 95°C, 36 cycles of 50 s at 94°C, 50 s of annealing at 57°C for the two chloroplast DNA fragments and 58°C for the nrITS region, and 50 s at 72°C, with a final 7 min extension at 72°C. PCR products were purified using a TIAN quick Midi Purification Kit following the recommended protocol (TIANGEN, Beijing, China). Sequencing reactions were performed with PCR primers covering the whole PCR segment using an ABI Prism Bigdye™ Terminator Cycle Sequencing Ready Reaction Kit. These PCR products were separated and visualized on an ABI 3130xl Genetic Analyser (Applied Biosystems). We further analyzed the sequence variation between different ITS copies that had additive peaks when directly sequenced. We ligated these PCR products into a pMD18-T vector (TaKaRa) according to the manufacturer's instructions, and propagated it in the JM109 strain of *Escherichia coli.* We then harvested and cultured positive clones and, finally, extracted plasmid DNA by using a U-gene Plasmid Mini Kit (U-gene) and sequenced them using the universal M13 primer.

We selected eight pairs of SSR primers for our population genetic data analyses [59]–[60]. Amplification reactions were performed in a volume of 20μl containing: 1×reaction buffer, 8–12 ng genomic DNA, 200 µM of each dNTP, 16 µM of each primer, and one unit of Taq DNA polymerase (TaKaRa). The Cycler^TM^ Thermal Cycler (Bio-Rad USA) PCR was programmed according to the following profile: 94°C for 7 min; 94°C for 30 s, 50–58°C for 30 s, 72°C for 30 s, 37 cycles; and 72°C for 7 min. The PCR products were resolved on 6.5% polyacrylamide denaturing gel and visualized by silver-staining. The band size was examined using a 10-bp DNA ladder (TaKaRa) as a reference.

### Data Analysis

We aligned the cpDNA and ITS sequences using Clustal X [61]. A matrix of combined cpDNA sequences was constructed for all 290 individuals, in which 11 different cpDNA sequences (chlorotypes) had been identified. Among the six nrITS genotypes, four had additive peaks of 1 to 2 sites, and were recombinants of the two other genotypes that were species-specific. These chloroplast fragments and ITS sequences have been deposited in GenBank under accession numbers JN162365–JN162386.

The relationships among both chlorotypes and nrITS genotypes were analyzed using the computer program NETWORK version 4.2.0.1 [62]. In the analysis of chlorotypes, both single site mutations and indels were assumed to have evolved with equal probability and each indel was assumed to have originated independently of other indels. Average gene diversity within populations (*H*_S_), total gene diversity (*H*_T_) and two measures of population differentiation, *G*_ST_ [63] and *N*_ST_, were calculated using the program PERMUT [64]. We inferred phylogeographic structure by testing whether *N*_ST_ was significantly larger than *G*_ST_ using a permutation test with 1,000 random permutations of haplotypes across populations. Genetic variation was examined between and within the two species by analysis of molecular variance (AMOVA) as implemented in ARLEQUIN version 3.0 [65], with significance tests based on 1,000 permutations.

To examine genetic differentiation and gene flow between the two species at the eight microsatellite loci, we conducted four independent analyses. First, we calculated expected heterozygosity (*H*_e_), observed heterozygosity (*H*_o_) and the effective number of alleles (*N*_e_) for each population using the program GENEPOP 3.4 [66]–[67] and we examined the distribution of genetic variation between and within species using an AMOVA analysis implemented in Arlequin 3.0 [68]. Second, we used GENECLASS version 2 to assign sampled individuals to either of the two species, assuming either a stepwise mutation model (SMM) or an infinite allele model (IAM) [69]. Third, we used STRUCTURE version 2.3.2 [70]–[71] to detect cryptic population structure and putative hybrids. This model assumes that the genome of individuals is a mixture of genes originating from *K* unknown ‘ancestral’ populations that may have undergone introgression events. We set each run to consist of a burn-in of 100,000 steps followed by 1,000,000 MCMC (Monte Carlo Markov Chain) replicates. To assess stability, 20 independent simulations were run for each value of *K* (from 1 to 10). To estimate the most likely number of clusters, the posterior probability of K, *L*(*K*) and Δ*K* were computed, using the runs with the highest probability for each *K*. Finally, we estimated the scaled mutation rates, θ, of the two species (4*N*_e_μ, where *N*_e_ = effective population size and µ = mutation rate) and the effective number of migrants (2*N*_e_m, where *N*_e_ is the effective population size and m is the migration rate) per generation using the software package MIGRATE version 3.2.6 [72]–[73]. We adopted the mutation rate of 10^−3^ per gamete per generation in poplars, as suggested by Lexer et al. [9]. Here, we assumed that all alleles shared between species at each locus resulted from gene flow, rather than from incomplete lineage sorting (ancestral polymorphisms). To avoid the confounding effects of differences in sample size on the estimate of gene flow, we picked a random subset of individuals from the larger group, with the number of individuals in the subset the same as in the smaller group. We carried out these estimations based on 20 short chains (10,000 trees) and three long chains (1,000,000) with 10,000 trees discarded as the initial ‘burn-in’.

## Supporting Information

Figure S1 **Phylogenetic tree of the twelve species in** ***Populus.*** This tree was constructed based on four cpDNA fragments (*trn*V, *trn*L, *mat*K and *rbc*L) by means of Bayesian analyses implemented in MrBayes version 3.1.2 (Huelsenbeck & Ronquist, 2001). The best model (GTR+I+R) was used and one cold and three heated chains were started from random initial trees and run for 6,000,000 generations, with sampling every 200 generations. After a burn-in period of the first 2,000,000 generations, 20,000 trees were sampled from the posterior distribution, and a majority rule consensus of these was generated to provide posterior probability scores for all nodes. Numbers above the branches are posterior probabilities supporting the corresponding branch when greater than 50%.
(TIF)

Figure S2 **Bayesian consensus tree from ITS. **This tree was constructed based on the ITS sequence matrix by means of Bayesian analyses implemented in MrBayes version 3.1.2 (Huelsenbeck & Ronquist, 2001). The best model (GTR+I+R) was used and one cold and three heated chains were started from random initial trees and run for 6,000,000 generations, with sampling every 200 generations. After a burn-in period of the first 2,000,000 generations, 20,000 trees were sampled from the posterior distribution, and a majority rule consensus of these was generated to provide posterior probability scores for all nodes. Numbers above the branches are posterior probabilities supporting the corresponding branch when greater than 50%.
(TIF)

Figure S3 **Morphological comparisons between** ***P. euphratica*** **and** ***P. pruinosa*** **.** A1. Dense hairs in *P. pruinosa*; B1. few hairs in *P. euphratica*. A2. The seedling leaves of *P. pruinosa* are ovate or kidney-shaped. B2. The seedling leaves of *P. euphratica* are lanceolate.
(TIF)

Figure S4 **Phylogenetic trees of 11 chlorotypes recovered from** ***P. euphratica*** **and** ***P. pruinosa*** **when** ***P. trichocarpa*** **was used as an outgroup.** Bayesian analyses implemented in MrBayes version 3.1.2 (Huelsenbeck & Ronquist, 2001) were used. The best model (GTR+I+R) was used and one cold and three heated chains were started from random initial trees and run for 6,000,000 generations, with sampling every 200 generations. After a burn-in period of the first 2,000,000 generations, 20,000 trees were sampled from the posterior distribution, and a majority rule consensus of these was generated to provide posterior probability scores for all nodes. Numbers above the branches are posterior probabilities supporting the corresponding branch when greater than 50%.
(TIF)

Figure S5 **Bayesian inference of the number of clusters (** ***K*** **) of the two poplar species.** (A) Mean *L*(*K*) for 20 replicate runs at each level of *K* proposed clusters. (B) *K* was estimated using the distribution of Δ*K* (second order rate of change of the likelihood distribution).
(TIF)

Figure S6 **The shared and specific proportions within species. **The proportions of the examined individuals with species-specific versus shared genotypes in the two species, using cpDNA, ITS and all SSR loci as markers.
(TIF)

Table S1 **List of the sampled individuals for** ***P. euphratica*** **and** ***P. pruinosa*** **with their locations and altitudes.**
(DOC)

Table S2 **Variable sites of the aligned sequences of two chloroplast DNA fragments in eleven haplotypes of** ***P. euphratica*** **and** ***P. pruinosa*** **(⋄, ⋆, □ indel).**
(DOC)

Table S3 **Three single nucleotide substitutions are distinguished between** ***P. euphratica*** **(Pe) and** ***P. pruinosa*** **(Pp).**
(DOC)

Table S4 **Estimates were obtained based on eight SSR loci using Analyses of molecular variance (AMOVA).**
(DOC)

Table S5 **Characteristics of eight polymorphic microsatellite loci for** ***P. euphratica*** **and** ***P. pruinosa.***
(DOC)

Table S6 **Genetic variation within populations of** ***P. euphratica*** **and** ***P. pruinosa*** **based on eight SSR loci.**
(DOC)
