# Supplementary figures and images for: Genetic Differentiation and Delimitation between Ecologically Diverged Populus euphratica and P. pruinosa
Source: PLoS One. 2011 Oct 19;6(10):e26530. doi: 10.1371/journal.pone.0026530 (PMC3197521; doi:10.1371/journal.pone.0026530)

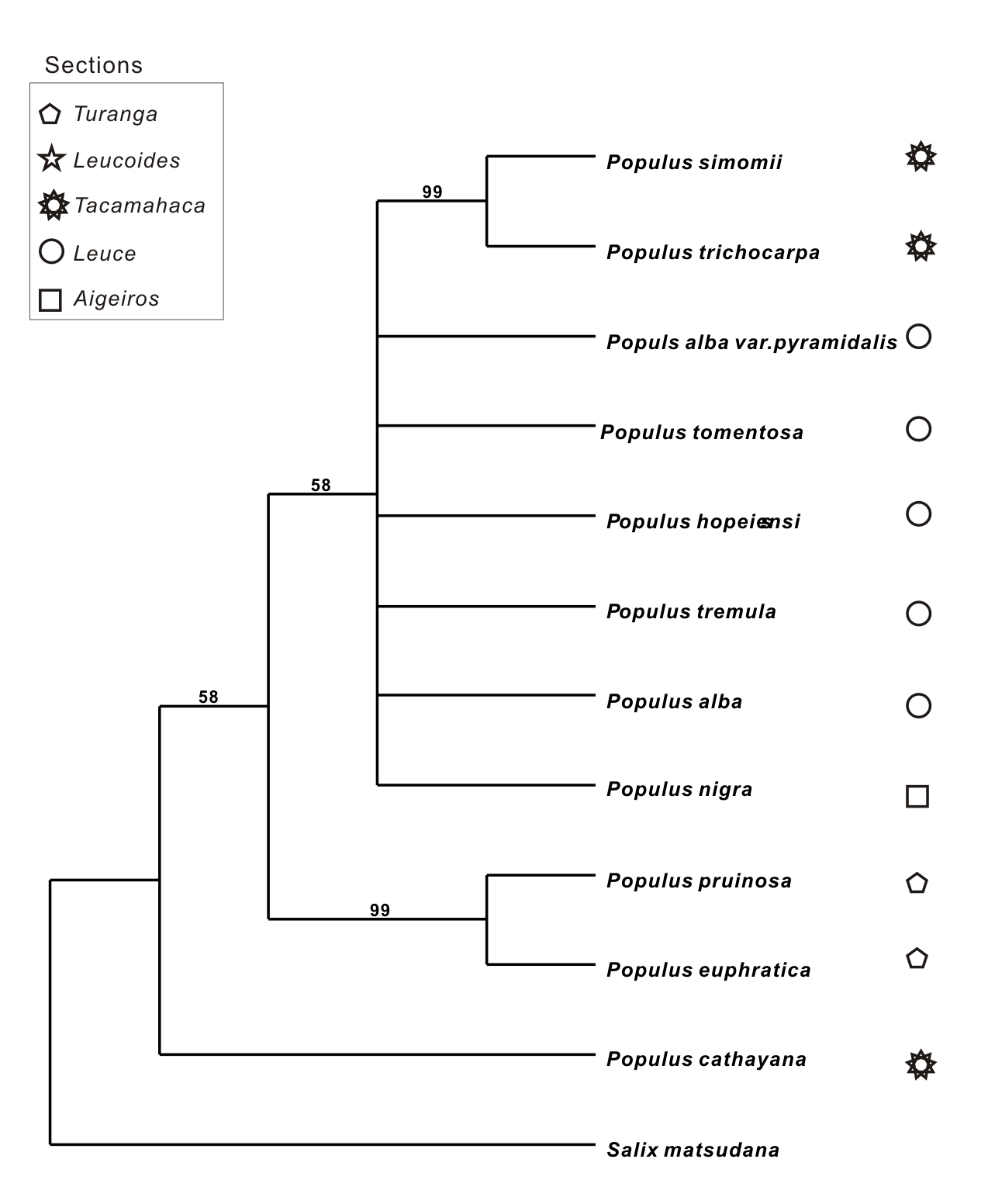

Supplement: Figure S1 — Phylogenetic tree of the twelve species in Populus. This tree was constructed based on four cpDNA fragments (trnV, trnL, matK and rbcL) by means of Bayesian analyses implemented in MrBayes version 3.1.2 (Huelsenbeck & Ronquist, 2001). The best model (GTR+I+R) was used and one cold and three heated chains were started from random initial trees and run for 6,000,000 generations, with sampling every 200 generations. After a burn-in period of the first 2,000,000 generations, 20,000 trees were sampled from the posterior distribution, and a majority rule consensus of these was generated to provide posterior probability scores for all nodes. Numbers above the branches are posterior probabilities supporting the corresponding branch when greater than 50%. (TIF) [file pone.0026530.s001.tif]

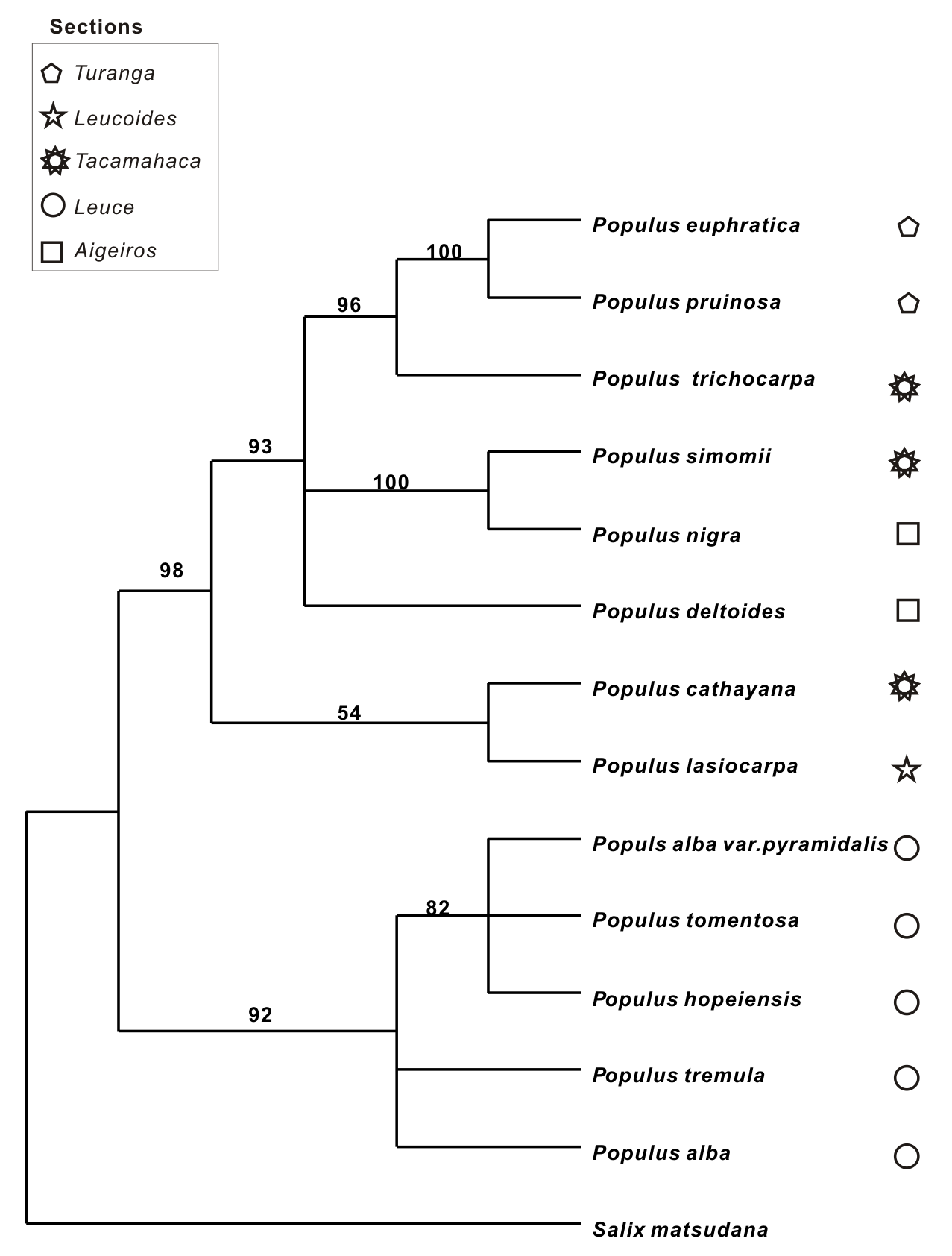

Supplement: Figure S2 — Bayesian consensus tree from ITS. This tree was constructed based on the ITS sequence matrix by means of Bayesian analyses implemented in MrBayes version 3.1.2 (Huelsenbeck & Ronquist, 2001). The best model (GTR+I+R) was used and one cold and three heated chains were started from random initial trees and run for 6,000,000 generations, with sampling every 200 generations. After a burn-in period of the first 2,000,000 generations, 20,000 trees were sampled from the posterior distribution, and a majority rule consensus of these was generated to provide posterior probability scores for all nodes. Numbers above the branches are posterior probabilities supporting the corresponding branch when greater than 50%. (TIF) [file pone.0026530.s002.tif]

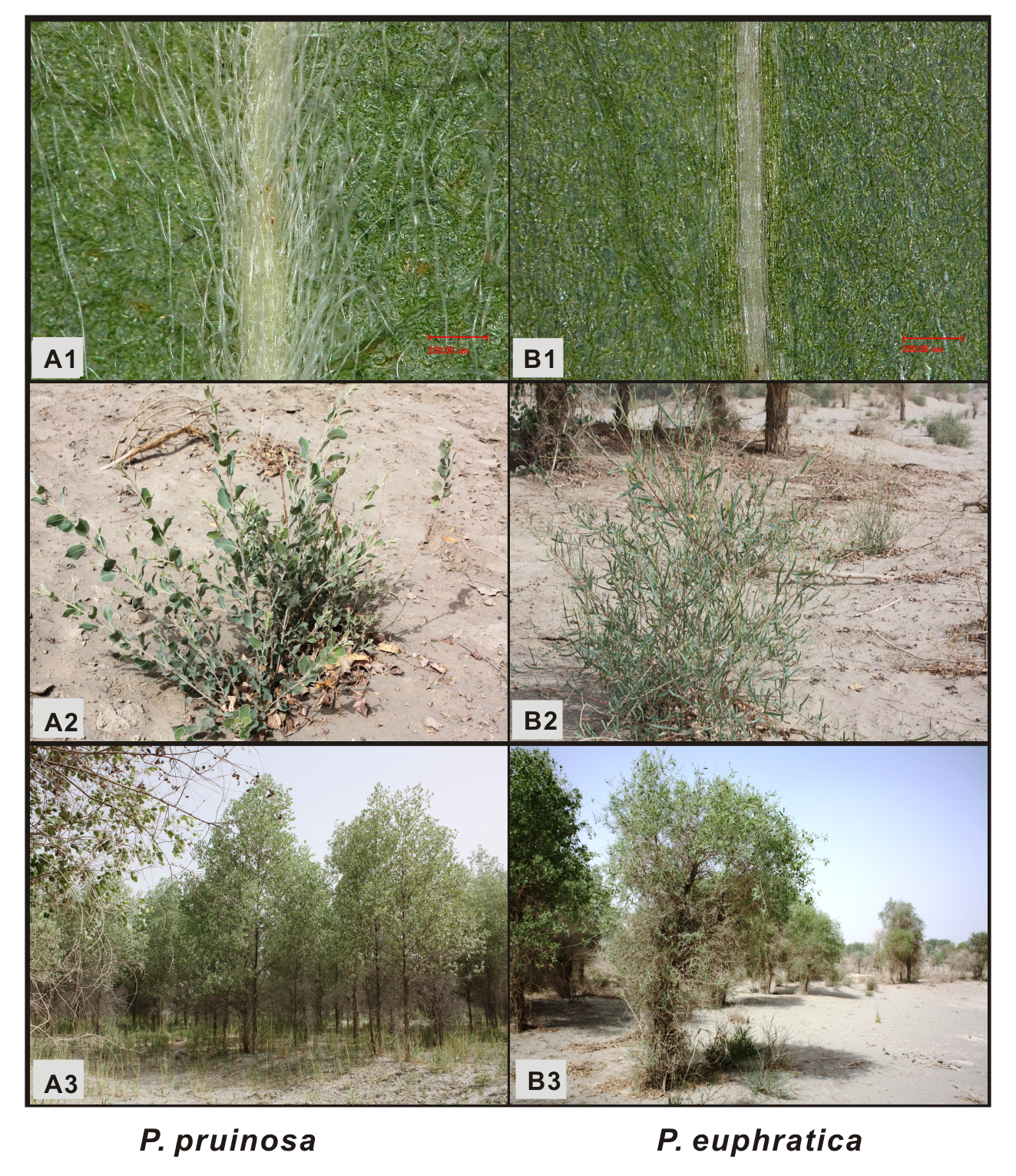

Supplement: Figure S3 — Morphological comparisons between P. euphratica and P. pruinosa . A1. Dense hairs in P. pruinosa; B1. few hairs in P. euphratica. A2. The seedling leaves of P. pruinosa are ovate or kidney-shaped. B2. The seedling leaves of P. euphratica are lanceolate. (TIF) [file pone.0026530.s003.tif]

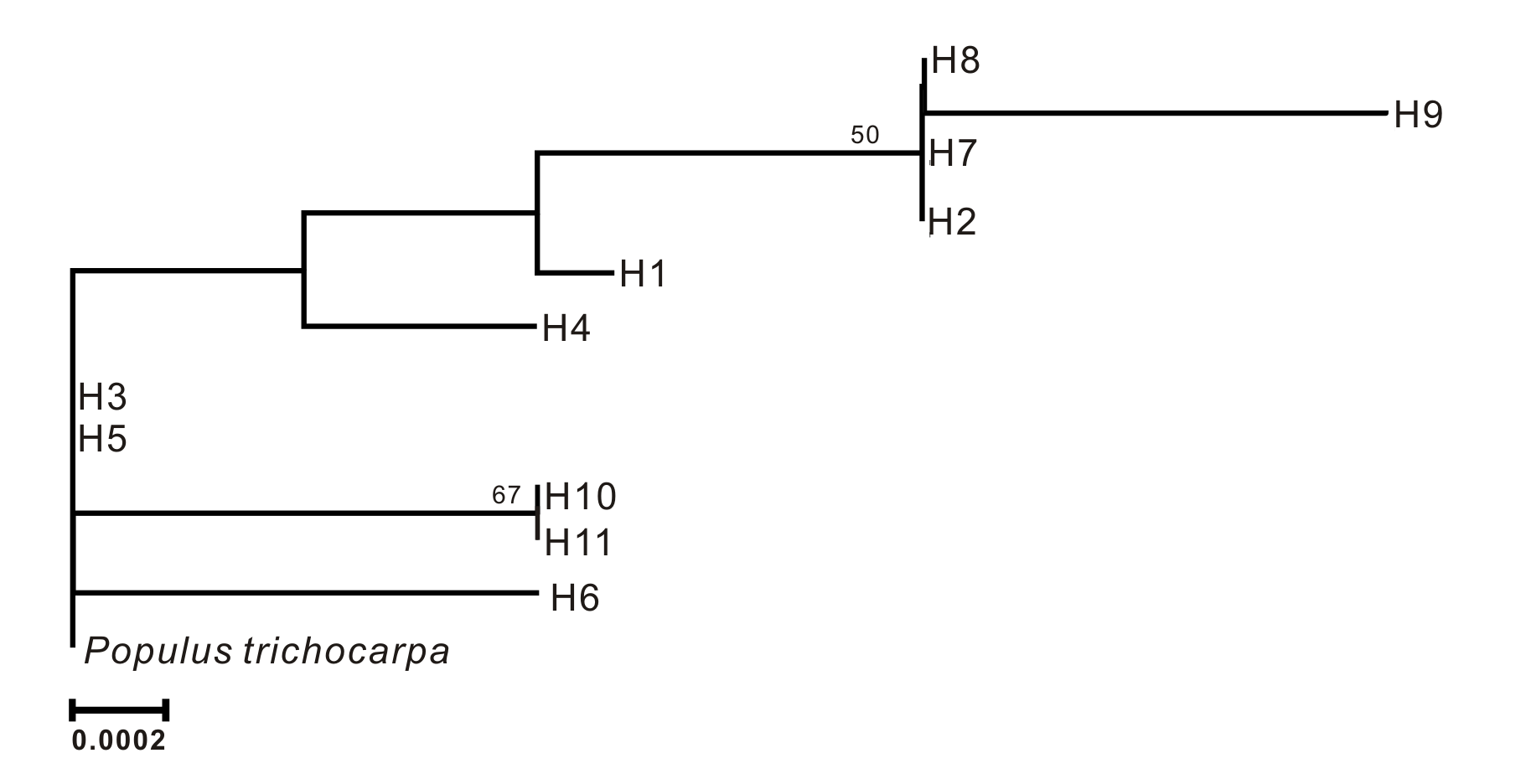

Supplement: Figure S4 — Phylogenetic trees of 11 chlorotypes recovered from P. euphratica and P. pruinosa when P. trichocarpa was used as an outgroup. Bayesian analyses implemented in MrBayes version 3.1.2 (Huelsenbeck & Ronquist, 2001) were used. The best model (GTR+I+R) was used and one cold and three heated chains were started from random initial trees and run for 6,000,000 generations, with sampling every 200 generations. After a burn-in period of the first 2,000,000 generations, 20,000 trees were sampled from the posterior distribution, and a majority rule consensus of these was generated to provide posterior probability scores for all nodes. Numbers above the branches are posterior probabilities supporting the corresponding branch when greater than 50%. (TIF) [file pone.0026530.s004.tif]

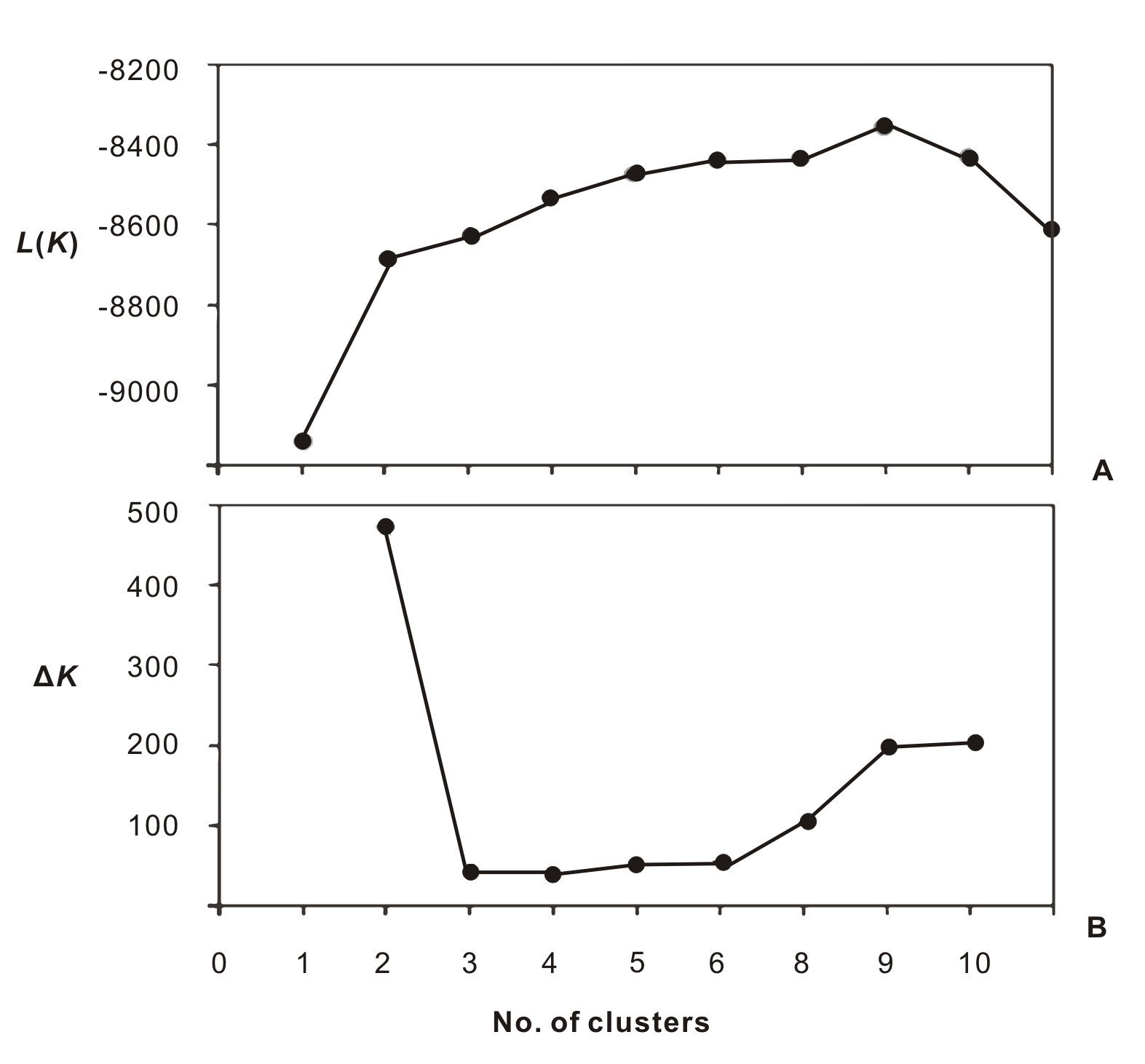

Supplement: Figure S5 — Bayesian inference of the number of clusters ( K ) of the two poplar species. (A) Mean L(K) for 20 replicate runs at each level of K proposed clusters. (B) K was estimated using the distribution of ΔK (second order rate of change of the likelihood distribution). (TIF) [file pone.0026530.s005.tif]

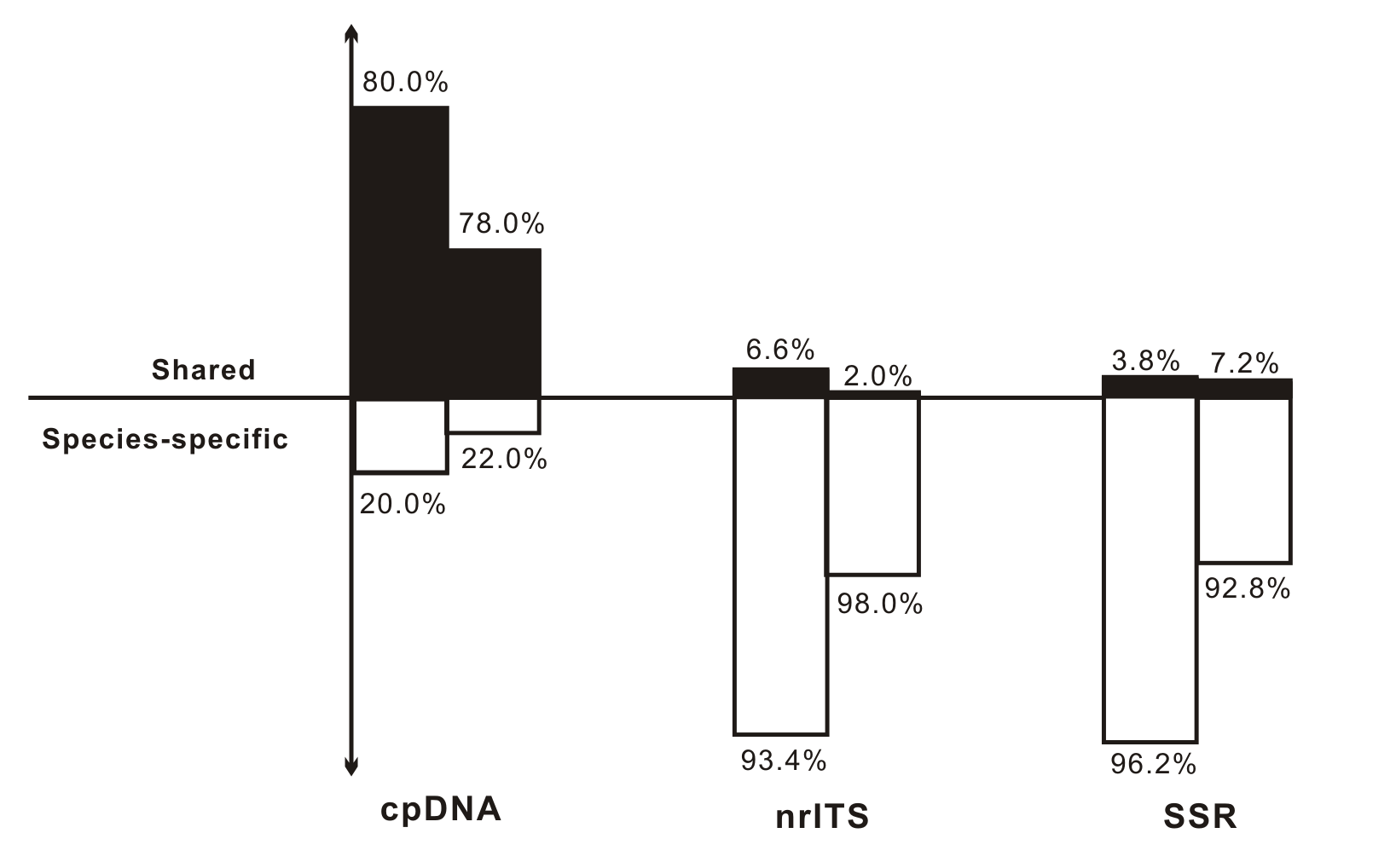

Supplement: Figure S6 — The shared and specific proportions within species. The proportions of the examined individuals with species-specific versus shared genotypes in the two species, using cpDNA, ITS and all SSR loci as markers. (TIF) [file pone.0026530.s006.tif]
