# Supplementary material for: Genetic Differentiation and Delimitation between Ecologically Diverged Populus euphratica and P. pruinosa
Source: PLoS One. 2011 Oct 19;6(10):e26530. doi: 10.1371/journal.pone.0026530 (PMC3197521; doi:10.1371/journal.pone.0026530)
Supplement: Table S1 — List of the sampled individuals for P. euphratica and P. pruinosa with their locations and altitudes. (DOC) [file pone.0026530.s007.doc]

**Table S1** List of the sampled individuals for *P. euphratica* and *P. pruinosa* with their locations and altitudes.

| **Species** | **Pop.** | **Location** | **Latitude (N)** | **Longitude (E)** | **Alt. (m)** |
| --- | --- | --- | --- | --- | --- |
| *P. euphratica* (n=200) | 1 | Akesu, XJ | 40°40′ | 81°44′ | 986 |
|  | 2 | Akesu, XJ | 40°27′ | 80°59′ | 1010 |
|  | 3 | Awati, XJ | 40°17′ | 80°21′ | 1030 |
|  | 4 | Akesu, XJ | 40°20′ | 79°56′ | 1040 |
|  | 5 | Bachu, XJ | 39°38′ | 78°24′ | 1120 |
|  | 6 | Maigaiti, XJ | 39°21′ | 78°05′ | 1140 |
|  | 7 | Shache, XJ | 38°23′ | 77°22′ | 1200 |
|  | 8 | Moyu, XJ | 37°34′ | 79°38′ | 1280 |
|  | 9 | Cele, XJ | 37°00′ | 80°40′ | 1340 |
|  | 10 | Minfeng,XJ | 37°11′ | 82°47′ | 1360 |
|  | 11 | Taqirang, XJ | 38°28′ | 85°43′ | 1130 |
|  | 12 | Kaogan, XJ | 39°44′ | 88°24′ | 802 |
|  | 13 | Kaogan, XJ | 40°35′ | 87°47′ | 840 |
|  | 14 | Lunnan, XJ | 41°15′ | 84°12′ | 918 |
|  | 15 | Baichen, XJ | 42°06′ | 83°08′ | 1750 |
|  | 16 | Shawan, XJ | 44°50′ | 85°29′ | 308 |
|  | 17 | Kelamayi, XJ | 46°08′ | 85°38′ | 302 |
|  | 18 | Ejinaqi, NMG | 42°01′ | 101°03′ | 302 |
|  | 19 | Baohuqu, NMG | 42°01′ | 101°14′ | 920 |
|  | 20 | Minqin, GS | 38°35′ | 102°58′ | 1367 |
| *P. pruinosa* (n=90) | 21(1) | Akesu, XJ | 40°42′ | 81° 40′ | 985 |
|  | 22(2) | Akesu, XJ | 40°26′ | 80°56′ | 1008 |
|  | 23(3) | Awati, XJ | 40°19′ | 80°15′ | 1023 |
|  | 24(4) | Akesu, XJ | 40°22′ | 79°59′ | 1038 |
|  | 25(5) | Bachu, XJ | 39°40′ | 78°25′ | 1119 |
|  | 26(6) | Maigaiti, XJ | 39°21′ | 78°03′ | 1140 |
|  | 27(7) | Shache, XJ | 38°25′ | 77°21′ | 1198 |
|  | 28(8) | Moyu, XJ | 37°36′ | 79°38′ | 1275 |
|  | 29(14) | Lunnan, XJ | 41°16′ | 87°49′ | 917 |

The number in brackets indicates the population number of the other species collected at the sympatric site.

Abbreviations: *n*, number of trees analyzed; Alt., altitude; GS, Gansu; XJ, XinJiang Autonomous Region; NX, Ningxia Hui Autonomous Region; NMG, NeiMenggu Autonomous Region.
