# Supplementary material for: Genetic Differentiation and Delimitation between Ecologically Diverged Populus euphratica and P. pruinosa
Source: PLoS One. 2011 Oct 19;6(10):e26530. doi: 10.1371/journal.pone.0026530 (PMC3197521; doi:10.1371/journal.pone.0026530)
Supplement: Table S2 — Variable sites of the aligned sequences of two chloroplast DNA fragments in eleven haplotypes of P. euphratica and P. pruinosa (⋄, ⋆, □ indel). (DOC) [file pone.0026530.s008.doc]

**Table S2** variable sites of the aligned sequences of two chloroplast DNA fragments in eleven haplotypes of *P. euphratica* and *P. pruinosa* (◇, *, □ indel).

| **Chlorotype** | ***Trn*V** | | | | | ***Trn*L** | | | |
| --- | --- | --- | --- | --- | --- | --- | --- | --- | --- |
|  | 35 | 238 | 262 | 466 | 519 | 76 | 221 | 341 | 359 |
| **H1** | C | ◇ | - | G | - | G | T | A | □ |
| **H2** | C | ◇ | - | G | - | G | T | T | □ |
| **H3** | C | ◇ | - | G | - | C | T | A | □ |
| **H4** | C | ◇ | - | G | - | C | T | T | □ |
| **H5** | C | - | - | G | - | C | T | A | □ |
| **H6** | A | ◇ | - | G | - | C | T | A | □ |
| **H7** | C | ◇ | - | G | - | G | T | T | - |
| **H8** | C | ◇ | ◇ | G | - | G | T | T | □ |
| **H9** | C | ◇ | - | A | - | G | T | T | □ |
| **H10** | C | ◇ | - | G | - | C | G | A | □ |
| **H11** | C | ◇ | - | G | * | C | G | A | □ |

◇, TTTCATATTGGATTATTTAAGTAA;

*, TATAT;

□, GAAACACTTTTC
