# Supplementary material for: Genetic Differentiation and Delimitation between Ecologically Diverged Populus euphratica and P. pruinosa
Source: PLoS One. 2011 Oct 19;6(10):e26530. doi: 10.1371/journal.pone.0026530 (PMC3197521; doi:10.1371/journal.pone.0026530)
Supplement: Table S3 — Three single nucleotide substitutions are distinguished between P. euphratica (Pe) and P. pruinosa (Pp). (DOC) [file pone.0026530.s009.doc]

**Table S3** Three single nucleotide substitutions are distinguished between *P. euphratica* (Pe) and *P. pruinosa* (Pp).

| **Genotype N.** | **20** | **381** | **387** | **species** |
| --- | --- | --- | --- | --- |
| **G1** | G | A | T | Pp |
| **G2** | A | G | C | Pe |
| **G3** | R | A | Y | Pe |
| **--- G3^a^** | A | A | C |  |
| **--- G3^b^** | G | A | T |  |
| **G4** | A | A | C | Pe |
| **G5** | R | G | Y | Pe |
| **--- G5^a^** | A | G | C |  |
| **--- G5^b^** | G | G | T |  |
| **G6** | R | A | T | Pe / Pp |
| **--- G6^a^** | A | A | T |  |
| **--- G6^b^** | G | A | T |  |
