# Supplementary material for: Genetic Differentiation and Delimitation between Ecologically Diverged Populus euphratica and P. pruinosa
Source: PLoS One. 2011 Oct 19;6(10):e26530. doi: 10.1371/journal.pone.0026530 (PMC3197521; doi:10.1371/journal.pone.0026530)
Supplement: Table S4 — Estimates were obtained based on eight SSR loci using Analyses of molecular variance (AMOVA). (DOC) [file pone.0026530.s010.doc]

**Table S4** Estimates were obtained based on eight SSR loci using Analyses of molecular variance (AMOVA).

| **Loci** | **Pe2** | **Pe4** | **Pe5** | **Pe6** | **Pe7** | **Pe8** | **Pe9** | **Pe16** | **Mean** | **Median** |
| --- | --- | --- | --- | --- | --- | --- | --- | --- | --- | --- |
| ***F_CT_*** | 0.4322 | -0.0167 | 0.2681 | 0.0587 | 0.6035 | 0.4632 | 0.0353 | -0.0103 | 0.2261 | 0.1634 |
