# Supplementary material for: Genetic Differentiation and Delimitation between Ecologically Diverged Populus euphratica and P. pruinosa
Source: PLoS One. 2011 Oct 19;6(10):e26530. doi: 10.1371/journal.pone.0026530 (PMC3197521; doi:10.1371/journal.pone.0026530)
Supplement: Table S5 — Characteristics of eight polymorphic microsatellite loci for P. euphratica and P. pruinosa. (DOC) [file pone.0026530.s011.doc]

**Table S5** Characteristics of eight polymorphic microsatellite loci for *P. euphratica* and *P. pruinosa.*

| **Species** | **Locus** | ***H_o_*** | ***H_e_*** | ***F_is_*** | ***F_it_*** | ***F_st_*** | ***N_a_*** | ***A*** | ***N_m_*** |
| --- | --- | --- | --- | --- | --- | --- | --- | --- | --- |
| *P. euphratica* | Pe2 | 1.0000 | 0.8339 | -0.3033 | -0.2015 | 0.0782 | 10 | 5.9419 | 2.9487 |
|  | Pe4 | 0.9196 | 0.8767 | -0.2644 | -0.0525 | 0.1675 | 13 | 7.9664 | 1.2422 |
|  | Pe5 | 0.9797 | 0.8509 | -0.2550 | -0.1546 | 0.0800 | 11 | 6.6103 | 2.8737 |
|  | Pe6 | 1.0000 | 0.8718 | -0.2972 | -0.1495 | 0.1138 | 12 | 7.6621 | 1.9459 |
|  | Pe7 | 0.8316 | 0.8674 | -0.0499 | 0.0403 | 0.0860 | 13 | 7.4184 | 2.6582 |
|  | Pe8 | 0.8520 | 0.8853 | -0.1241 | 0.0354 | 0.1419 | 12 | 8.5492 | 1.5112 |
|  | Pe9 | 0.9898 | 0.7106 | -0.4676 | -0.3981 | 0.0473 | 8 | 3.4340 | 5.0307 |
|  | Pe16 | 1.0000 | 0.8274 | -0.3402 | -0.2115 | 0.0960 | 10 | 5.7232 | 2.3530 |
|  | **Mean** | 0.9466 | 0.8405 | -0.2587 | -0.1290 | 0.1030 | 11.13 | 6.6632 | 2.1766 |
| *P. pruinosa* | Pe2 | 1.0000 | 0.7238 | -0.4423 | -0.3912 | 0.0354 | 7 | 3.5661 | 6.8043 |
|  | Pe4 | 0.8764 | 0.893 | -0.1781 | 0.0236 | 0.1712 | 13 | 8.9301 | 1.2099 |
|  | Pe5 | 1.0000 | 0.8546 | -0.2578 | -0.1775 | 0.0638 | 12 | 6.6472 | 3.6682 |
|  | Pe6 | 0.1573 | 0.7550 | 0.6775 | 0.7803 | 0.3189 | 8 | 4.0127 | 0.5340 |
|  | Pe7 | 0.5581 | 0.7865 | 0.1148 | 0.2728 | 0.1785 | 11 | 4.5852 | 1.1508 |
|  | Pe8 | 0.4157 | 0.7364 | 0.3516 | 0.4363 | 0.1308 | 8 | 3.7346 | 1.6620 |
|  | Pe9 | 0.9767 | 0.7209 | -0.4717 | -0.3614 | 0.0749 | 6 | 3.5295 | 3.0872 |
|  | Pe16 | 0.2159 | 0.5414 | 0.4566 | 0.6109 | 0.2840 | 6 | 2.1659 | 0.6302 |
|  | **Mean** | 0.6500 | 0.7514 | -0.0268 | 0.1300 | 0.1528 | 8.88 | 4.6464 | 1.3866 |

*H*_o_, observed heterozygosity; *H*_e_, expected heterozygosity; *F*_is_, inbreeding coefficient at the population level; *F*_it_, inbreeding coefficient at the total sample level; *F*_st_, proportion of differentiation among populations; *N*m, gene flow estimated from *N*_m_=0.25*(1-*F*_st_)/*F*_st_; *A*, Effective number of alleles; *N*_a_, Observed number of alleles.
