# Supplementary material for: Genetic Differentiation and Delimitation between Ecologically Diverged Populus euphratica and P. pruinosa
Source: PLoS One. 2011 Oct 19;6(10):e26530. doi: 10.1371/journal.pone.0026530 (PMC3197521; doi:10.1371/journal.pone.0026530)
Supplement: Table S6 — Genetic variation within populations of P. euphratica and P. pruinosa based on eight SSR loci. (DOC) [file pone.0026530.s012.doc]

**Table S6** Genetic variation within populations of *P.* *euphratica* and *P. pruinosa* based on eight SSR loci.

| **Species** | **Population** | ***N*_at_** | ***N*_r_** | ***N*_e_** | ***H*_o_** | ***H*_e_** | ***H*_pop_** |
| --- | --- | --- | --- | --- | --- | --- | --- |
| *P. euphratica* | 1 | 11 | 0 | 4.6480 | 0.9500 | 0.8102 | 1.5887 |
|  | 2 | 12 | 1 | 5.2612 | 0.9750 | 0.8445 | 1.7602 |
|  | 3 | 13 | 2 | 6.6250 | 0.9875 | 0.8395 | 1.7235 |
|  | 4 | 12 | 1 | 4.9265 | 0.9625 | 0.8252 | 1.6809 |
|  | 5 | 11 | 0 | 4.4722 | 0.9722 | 0.8003 | 1.5349 |
|  | 6 | 10 | 0 | 4.1686 | 1.0000 | 0.7822 | 1.5276 |
|  | 7 | 12 | 1 | 3.7736 | 0.9625 | 0.8444 | 1.4582 |
|  | 8 | 13 | 2 | 5.1074 | 0.9250 | 0.8397 | 1.7732 |
|  | 9 | 10 | 1 | 4.0549 | 0.7750 | 0.7667 | 1.5425 |
|  | 10 | 13 | 1 | 4.6831 | 0.9500 | 0.7863 | 1.5533 |
|  | 11 | 10 | 0 | 4.4861 | 0.9611 | 0.7989 | 1.5466 |
|  | 12 | 11 | 1 | 4.2619 | 0.9306 | 0.7687 | 1.5308 |
|  | 13 | 11 | 0 | 4.7037 | 0.8778 | 0.8234 | 1.6463 |
|  | 14 | 12 | 1 | 4.9453 | 0.9500 | 0.8316 | 1.7064 |
|  | 15 | 11 | 2 | 3.4377 | 1.0000 | 0.7150 | 1.2613 |
|  | 16 | 11 | 1 | 3.3785 | 0.9250 | 0.7204 | 1.2997 |
|  | 17 | 10 | 0 | 4.1418 | 0.9875 | 0.7487 | 1.4153 |
|  | 18 | 11 | 0 | 4.2182 | 1.0000 | 0.7920 | 1.5145 |
|  | 19 | 12 | 2 | 3.6996 | 0.9500 | 0.7609 | 1.4175 |
|  | 20 | 11 | 1 | 3.9982 | 0.8875 | 0.7528 | 1.4737 |
|  | **Mean** | 10.90 | 0.95 | 3.6822 | 0.9349 | 0.7463 | 1.4737 |
| *P. pruinosa* | 21 | 12 | 1 | 3.8243 | 0.7958 | 0.7364 | 1.4204 |
|  | 22 | 13 | 0 | 4.1462 | 0.6625 | 0.7351 | 1.4393 |
|  | 23 | 11 | 3 | 3.1221 | 0.5750 | 0.5796 | 1.0937 |
|  | 24 | 13 | 2 | 3.8945 | 0.6625 | 0.6801 | 1.3126 |
|  | 25 | 11 | 0 | 2.9575 | 0.5000 | 0.5341 | 1.0296 |
|  | 26 | 10 | 1 | 3.8075 | 0.6500 | 0.7434 | 1.3948 |
|  | 27 | 11 | 1 | 2.9112 | 0.6389 | 0.6673 | 1.1557 |
|  | 28 | 11 | 1 | 2.6386 | 0.6125 | 0.5890 | 1.0290 |
|  | 29 | 11 | 0 | 3.4298 | 0.7625 | 0.7266 | 1.3124 |
|  | **Mean** | 10.36 | 0.50 | 2.8638 | 0.6371 | 0.6458 | 1.1217 |

*N*_at_, total number of alleles per population; *N*_r_, number of rare alleles (alleles present in fewer than 5% of the individuals) per population; *N*_e_, Effective number of alleles per population; *H*_o_, observed heterozygosity; *H*_e_, expected heterozygosity; *H*_pop_, Shannon’s information index.
